# Supplementary material for: ADAR1 Promotes Myogenic Proliferation and Differentiation of Goat Skeletal Muscle Satellite Cells
Source: Cells. 2024 Sep 25;13(19):1607. doi: 10.3390/cells13191607 (PMC11475720; doi:10.3390/cells13191607)
Supplement: Supplementary file 1 [file cells-13-01607-s001.zip › Supplementary Figures.pdf]

Supplementary Figures

ADAR1 Promotes Proliferation and Differentiation of Goat Muscle Satellite Cells

Zihao Zhao [†]**, Miao Xiao** [†]**, Xiaoli Xu, Meijun Song, Dinghui Dai, Siyuan Zhan, Jiaxue Cao, Jiazhong Guo, Tao Zhong, Linjie Wang, Li Li*** and **Hongping Zhang***

Farm Animal Genetic Resources Exploration Innovation Key Laboratory of Sichuan Province, College of Animal Science and Technology, Sichuan Agricultural University, Chengdu 611130, China
* Correspondence: lily@sicau.edu.cn (L.L.); zhp@sicau.edu.cn (H.Z.).
[†] These authors contributed equally to this work.

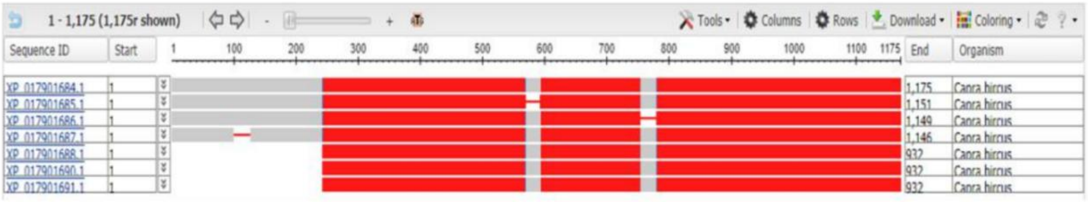

Figure S1. Alignment of amino acid of goat ADAR1 isoforms X1-X7.

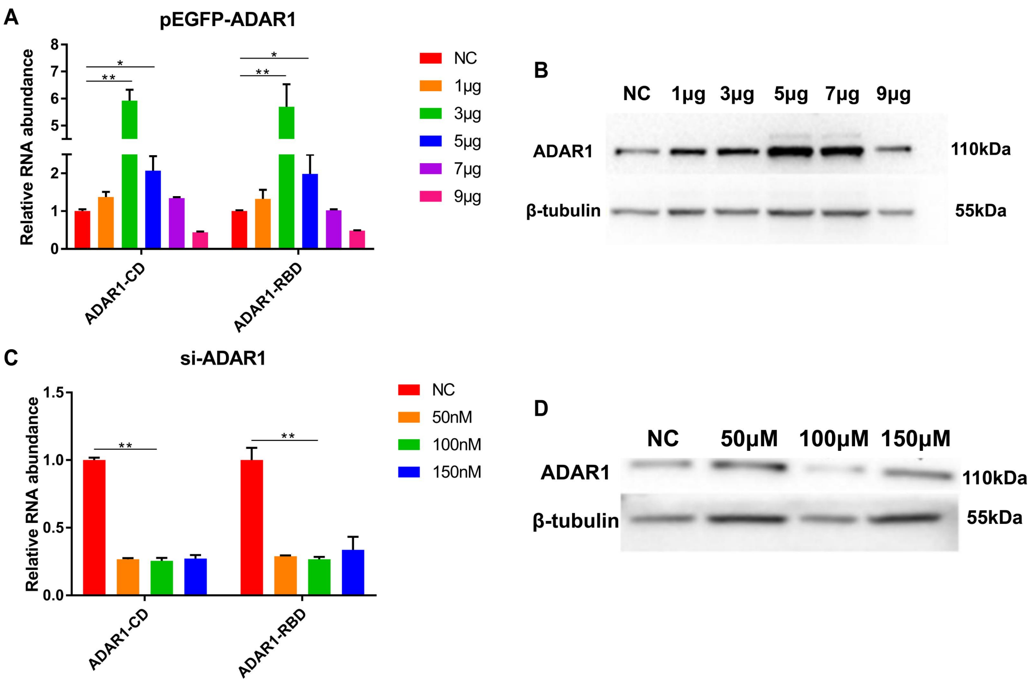

Figure S2. The optimization of overexpression and interfering ADAR1 in goat MuSCs. (A) The levels of ADAR1 catalytic domain (ADAR1-CD) and RNA binding domain (ADAR1-RBD) detected by RT-qPCR in cells transfected with pEGFP-ADAR1 plasmid. (B) The protein of ADAR1 after transfection with pEGFP-ADAR1 plasmid. (C) The levels of ADAR1-CD and ADAR1-RBD detected by RT-qPCR in cells treated with siRNA. (D) The protein of ADAR1 after interfering with its siRNA.

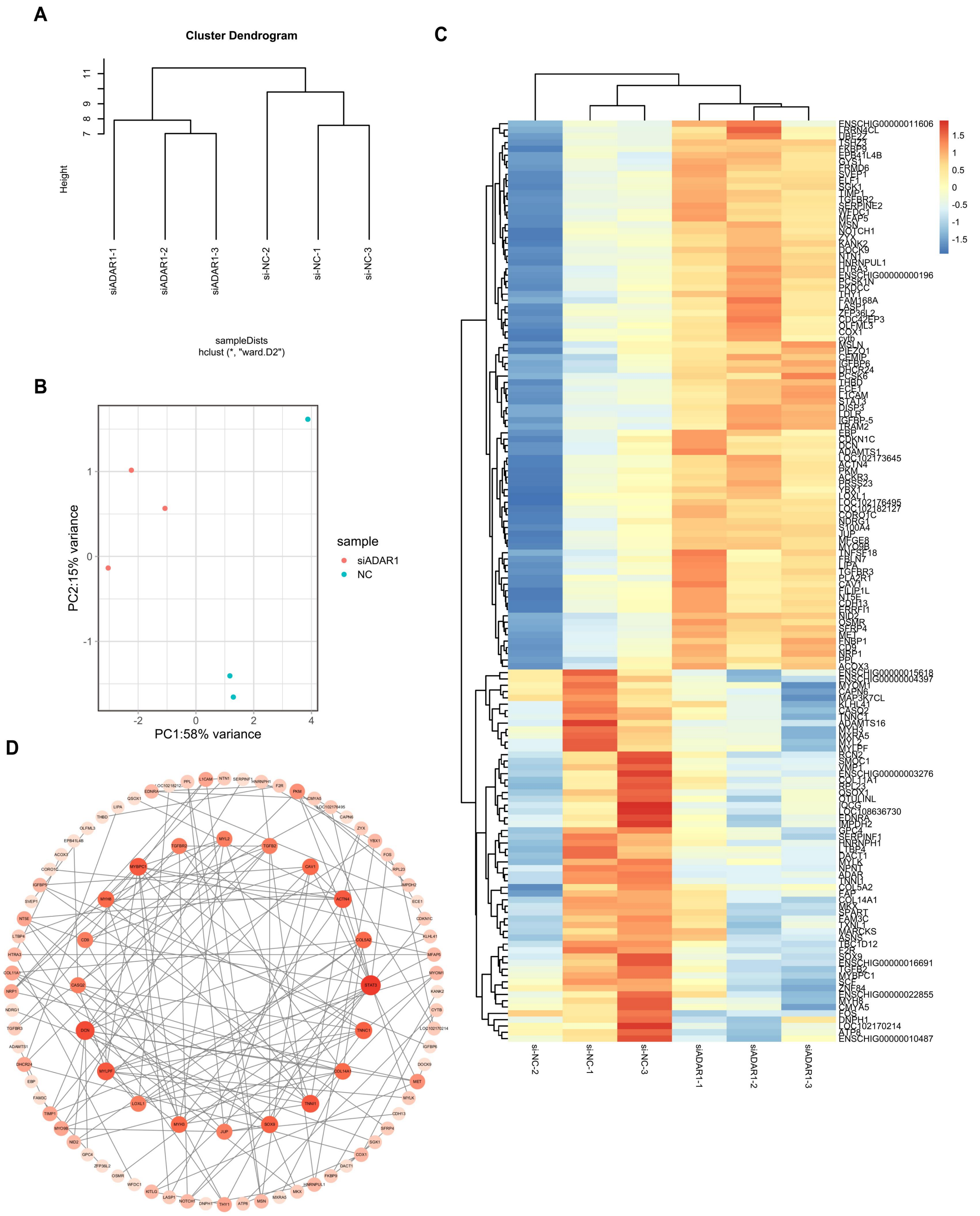

Figure S3. Genes affected by ADAR1 in myogenic differentiation of goat MuSCs. (A) Hierarchical clustering of RNA-seq samples. (B) PCA analysis of mRNA-seq data. (C)Heat map of DEGs. (D) The PPI network of DEGs protein.
