# Supplementary material for: ADAR1 Promotes Myogenic Proliferation and Differentiation of Goat Skeletal Muscle Satellite Cells
Source: Cells. 2024 Sep 25;13(19):1607. doi: 10.3390/cells13191607 (PMC11475720; doi:10.3390/cells13191607)
Supplement: Supplementary file 1 [file cells-13-01607-s001.zip › Supplementary Tables.pdf]

# Supplementary Tables

# ADAR1 Promotes Proliferation and Differentiation of Goat Muscle Satellite Cells

**Zihao Zhao [†], Miao Xiao [†], Xiaoli Xu, Meijun Song, Dinghui Dai, Siyuan Zhan, Jiaxue Cao, Jiazhong Guo, Tao Zhong, Linjie Wang, Li Li* and Hongping Zhang***

Farm Animal Genetic Resources Exploration Innovation Key Laboratory of Sichuan Province,
College of Animal Science and Technology, Sichuan Agricultural University, Chengdu 611130, China
* Correspondence: lily@sicau.edu.cn (L.L.); zhp@sicau.edu.cn (H.Z.).
[†] These authors contributed equally to this work.

## Table S1. Primer sequences information

| Gene name | Forward primer (5′-3′) | Reserve primer (5′-3′) | Tm (℃) | Amplicon size (bp) | GenBank accession | Function |
|---|---|---|---|---|---|---|
| ADAR1-CDS | GCTTGAGTCGACTCCAGAGGCG | CACTGGGCAAAGGTAA | 62 | 3510 | XM_018046195 | CDS cloning |
| ADAR1-EC | *Kpn1*-F: CGGGGTACCGCTTGAGTCGACTCCAGAGGCG | *BamH1*: CGCGGATCCCACTGGGCAAAGGTAA | 62 | 3528 | XM_018046195 | Plasmid construction |
| ADAR1-CD | TGAAATACAACCCCCAGACG | TTATCAAAGAGGGCACCATC | 61.4 | 139 | XM_018046195 | RT-qPCR |
| ADAR1-RBD | CAGATACCTGAACACCAACC | TCGTCCTCCCCAATCAAAAC | 64.5 | 231 | XM_018046195 | RT-qPCR |
| β-Actin | CCTGCGGCATTCACGAAACTAC | ACAGCACCGTGTTGGCGTAGAG | 59 | 87 | XM_018039831.1 | RT-qPCR |
| PAX7 | AGGACGAAGCGGACAAGAA | TCCAGACGGTTCCCTTTGT | 57 | 91 | XM_018054740.1 | RT-qPCR |
| PCNA | TGAAGAAAGTGCTGGAGGCG | TTTGGACATGCTGGTGAGG | 61.4 | 197 | XM_005688167.3 | RT-qPCR |
| MyoD | GTGCAAACGCAAGACGACTA | GCTGGTTTGGGTTGCTAGAC | 61.4 | 128 | XM_018058990.1 | RT-qPCR |
| MyoG | GGACCCTACAGATGCCCACAA | TTGGTATGGTTTCATCTGGG | 59 | 101 | NM_001285733.1 | RT-qPCR |
| MyHC | AGTCTTTGTGGCGGACCCTA | TTGGCTGTCACCTTCCGCC | 63.3 | 78 | XM_018064658.1 | RT-qPCR |
| Myomaker | CCCTGGCTCTCATGTTGCGCTT | TGCACTTCCGGCCTTCTTGTTG | 64.5 | 131 | XM_018056156.1 | RT-qPCR |
| Myomerger | GGGCTGTCTGTTGTTCGTCC | AGCATTTCAGGGGGCACAGC | 55.7 | 146 | XM_018038691.1 | RT-qPCR |

**Table S2. Quality summary of mRNA-seq data**

| Sample | Raw data | Clean data | Raw bases | Clean bases | Q20 (%) | Q30 (%) |
|---|---|---|---|---|---|---|
| siNC-1 | 27,882,987 | 26,574,180 | 8.36G | 7.97G | 97.41 | 92.75 |
| siNC-2 | 22,033,027 | 21,135,821 | 6.61G | 6.34G | 97.52 | 92.91 |
| siNC-3 | 29,135,427 | 27,858,612 | 8.74G | 8.36G | 97.47 | 92.88 |
| siADAR1-1 | 31,240,708 | 29,107,201 | 9.37G | 8.73G | 97.34 | 92.65 |
| siADAR1-2 | 27,122,116 | 26,107,295 | 8.14G | 7.83G | 97.27 | 92.38 |
| siADAR1-3 | 27,716,441 | 26,502,698 | 8.31G | 7.95G | 97.3 | 92.53 |
